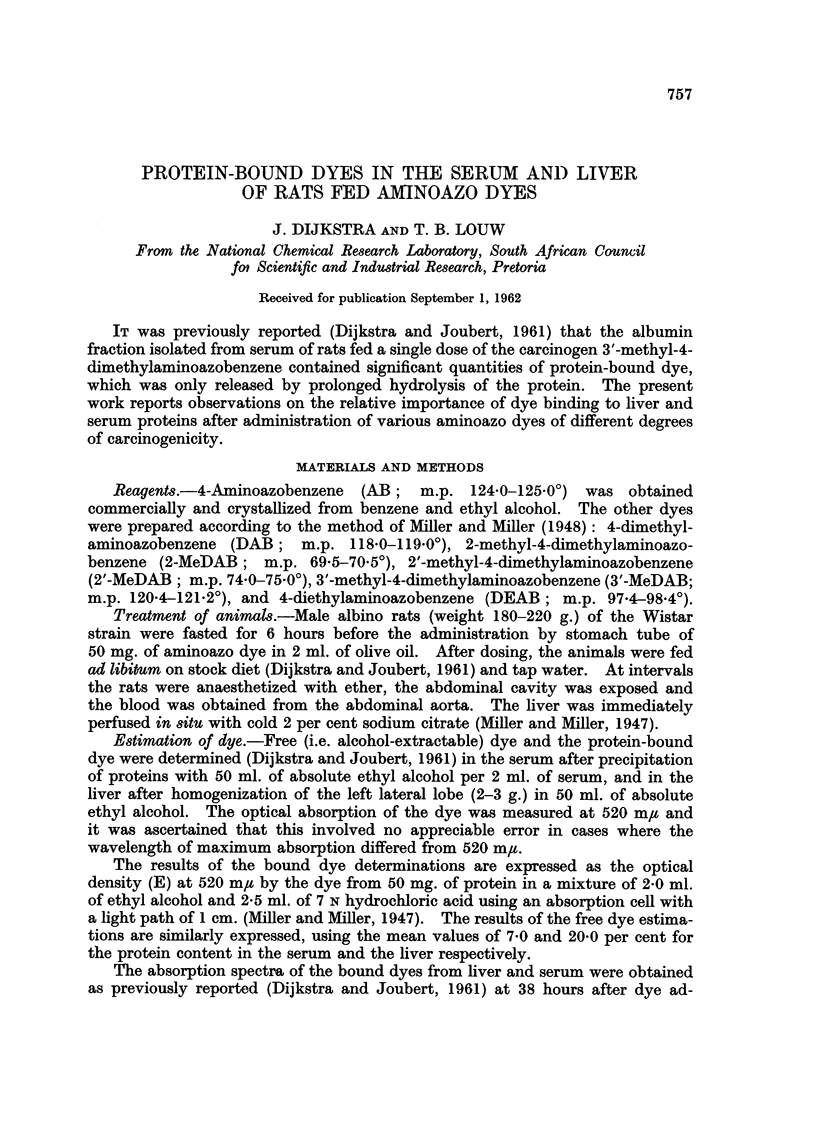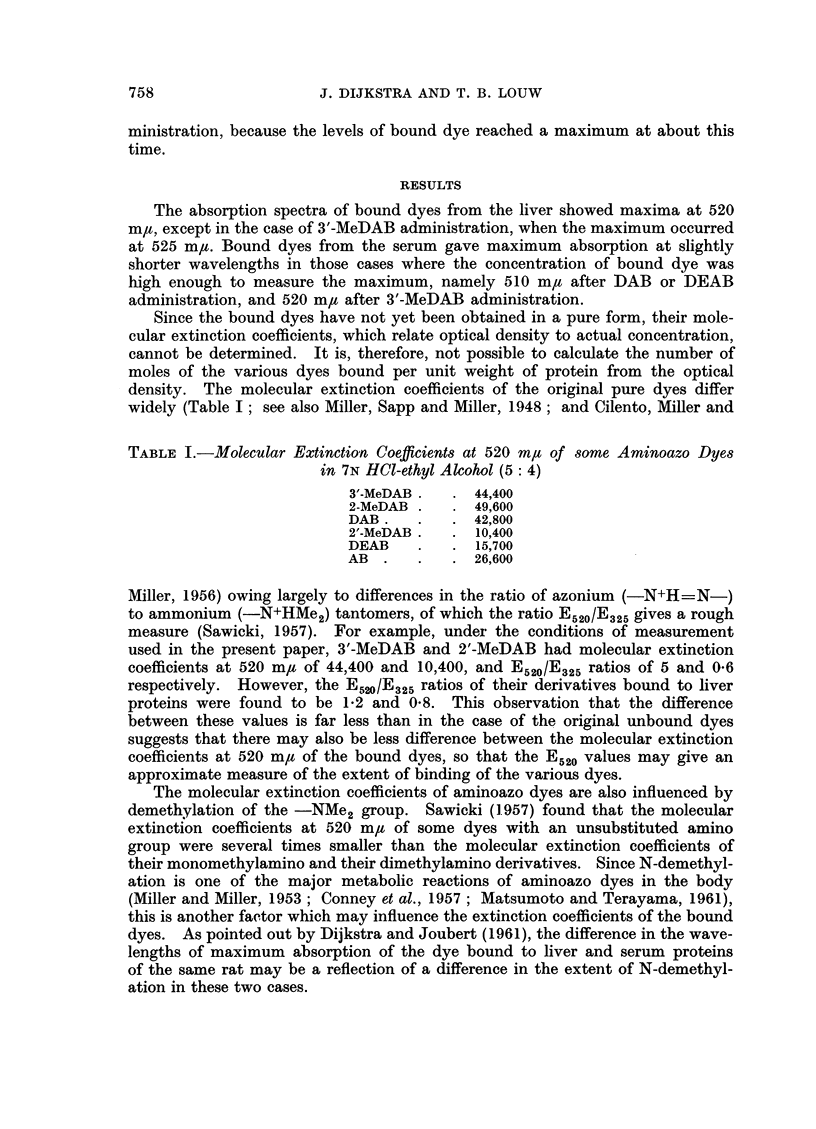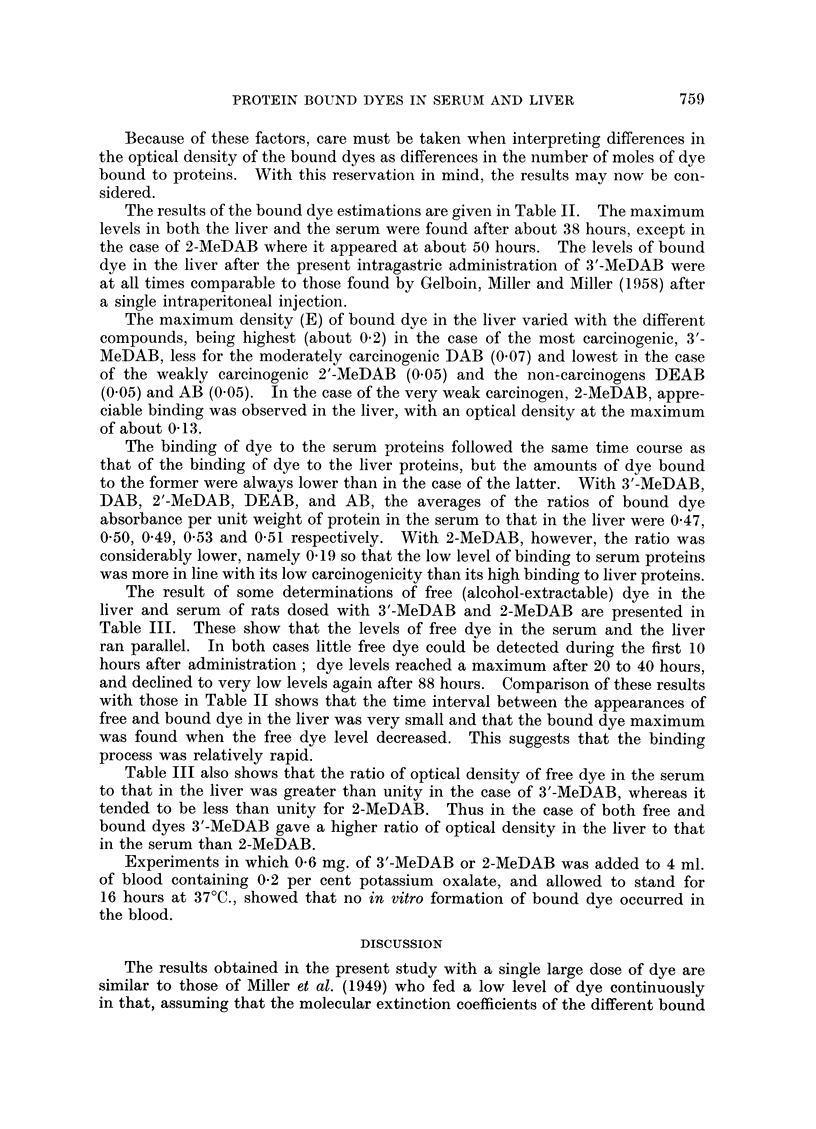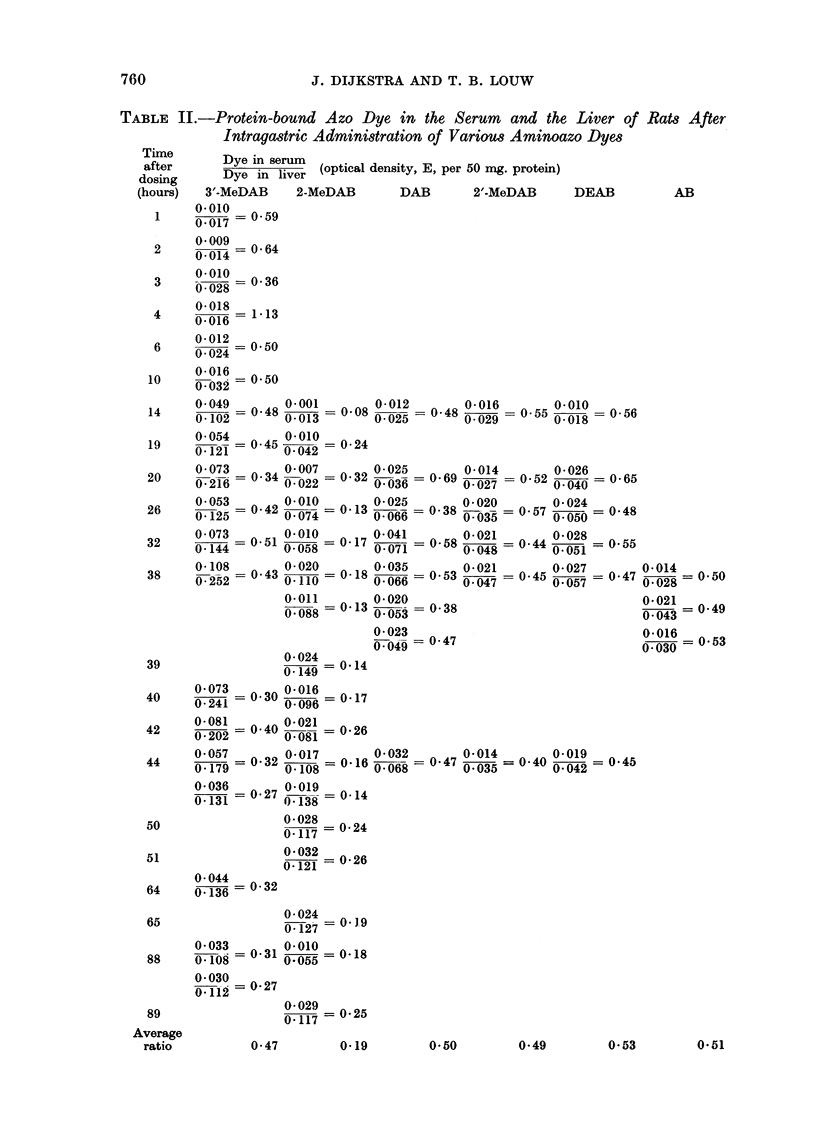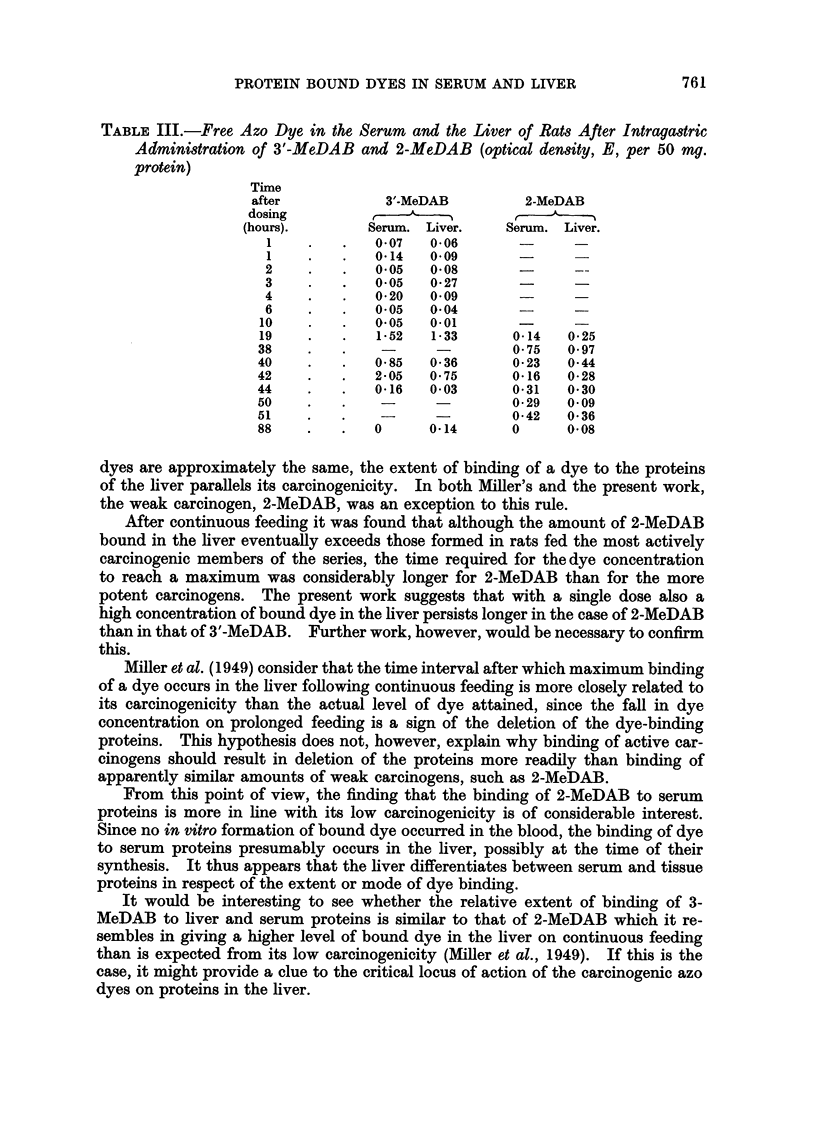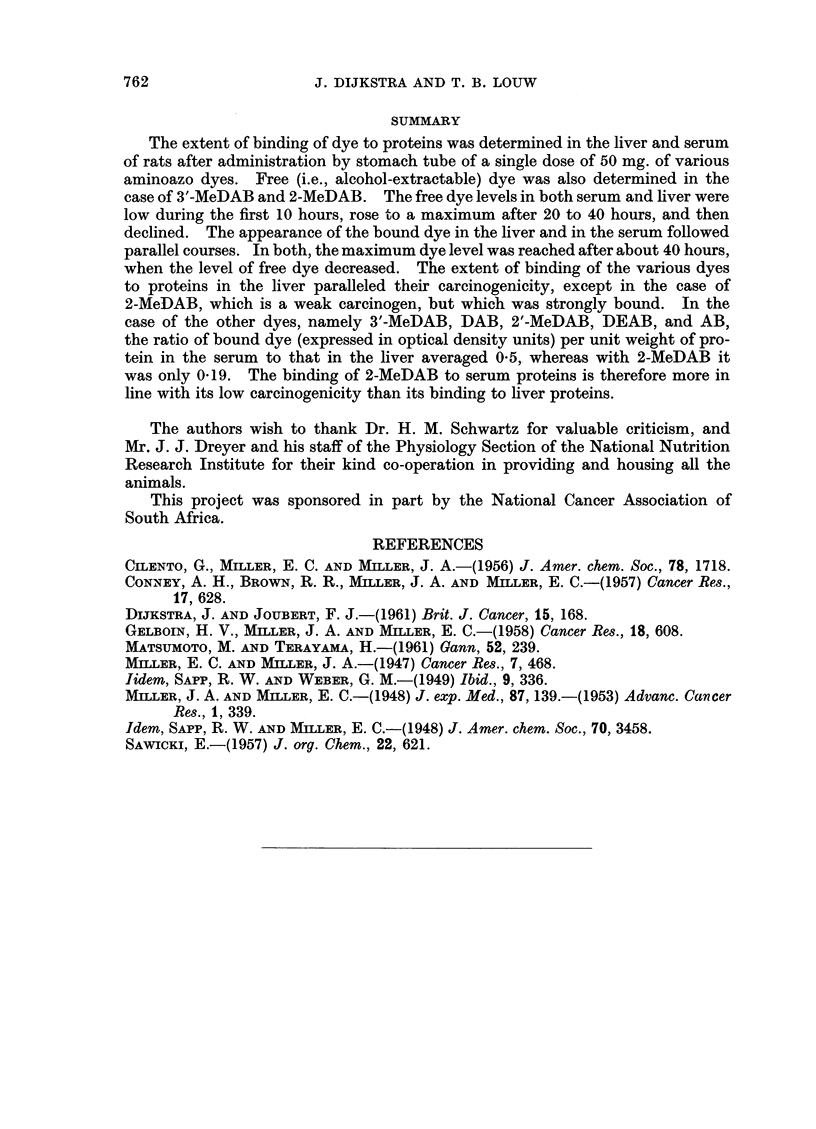# Protein-Bound Dyes in the Serum and Liver of Rats Fed Aminoazo Dyes

**DOI:** 10.1038/bjc.1962.88

**Published:** 1962-12

**Authors:** J. Dijkstra, T. B. Louw


### PROTEIN-BOUND DYES IN THE SERUM AND LIVER OF RATS FED AMINOAZO DYES

J. DIJKSTRA AND T. B. LOUW

*From the National Chemical Research Laboratory, South African Council for Scientific and Industrial Research, Pretoria*

Received for publication September 1, 1962

IT was previously reported (Dijkstra and Joubert, 1961) that the albumin fraction isolated from serum of rats fed a single dose of the carcinogen 3′-methyl-4-dimethylaminoazobenzene contained significant quantities of protein-bound dye, which was only released by prolonged hydrolysis of the protein. The present work reports observations on the relative importance of dye binding to liver and serum proteins after administration of various aminoazo dyes of different degrees of carcinogenicity.

### MATERIALS AND METHODS

*Reagents.*—4-Aminoazobenzene (AB ; m.p. 124·0–125·0°) was obtained commercially and crystallized from benzene and ethyl alcohol. The other dyes were prepared according to the method of Miller and Miller (1948) : 4-dimethylaminoazobenzene (DAB ; m.p. 118·0–119·0°), 2-methyl-4-dimethylaminoazobenzene (2-MeDAB ; m.p. 69·5–70·5°), 2′-methyl-4-dimethylaminoazobenzene (2′-MeDAB ; m.p. 74·0–75·0°), 3′-methyl-4-dimethylaminoazobenzene (3′-MeDAB; m.p. 120·4–121·2°), and 4-diethylaminoazobenzene (DEAB ; m.p. 97·4–98·4°).

*Treatment of animals.*—Male albino rats (weight 180–220 g.) of the Wistar strain were fasted for 6 hours before the administration by stomach tube of 50 mg. of aminoazo dye in 2 ml. of olive oil. After dosing, the animals were fed *ad libitum* on stock diet (Dijkstra and Joubert, 1961) and tap water. At intervals the rats were anaesthetized with ether, the abdominal cavity was exposed and the blood was obtained from the abdominal aorta. The liver was immediately perfused *in situ* with cold 2 per cent sodium citrate (Miller and Miller, 1947).

*Estimation of dye.*—Free (i.e. alcohol-extractable) dye and the protein-bound dye were determined (Dijkstra and Joubert, 1961) in the serum after precipitation of proteins with 50 ml. of absolute ethyl alcohol per 2 ml. of serum, and in the liver after homogenization of the left lateral lobe (2–3 g.) in 50 ml. of absolute ethyl alcohol. The optical absorption of the dye was measured at 520 m$\mu$ and it was ascertained that this involved no appreciable error in cases where the wavelength of maximum absorption differed from 520 m$\mu$.

The results of the bound dye determinations are expressed as the optical density (E) at 520 m$\mu$ by the dye from 50 mg. of protein in a mixture of 2·0 ml. of ethyl alcohol and 2·5 ml. of 7 N hydrochloric acid using an absorption cell with a light path of 1 cm. (Miller and Miller, 1947). The results of the free dye estimations are similarly expressed, using the mean values of 7·0 and 20·0 per cent for the protein content in the serum and the liver respectively.

The absorption spectra of the bound dyes from liver and serum were obtained as previously reported (Dijkstra and Joubert, 1961) at 38 hours after dye administration, because the levels of bound dye reached a maximum at about this time.

### RESULTS

The absorption spectra of bound dyes from the liver showed maxima at 520 m$\mu$, except in the case of 3'-MeDAB administration, when the maximum occurred at 525 m$\mu$. Bound dyes from the serum gave maximum absorption at slightly shorter wavelengths in those cases where the concentration of bound dye was high enough to measure the maximum, namely 510 m$\mu$ after DAB or DEAB administration, and 520 m$\mu$ after 3'-MeDAB administration.

Since the bound dyes have not yet been obtained in a pure form, their molecular extinction coefficients, which relate optical density to actual concentration, cannot be determined. It is, therefore, not possible to calculate the number of moles of the various dyes bound per unit weight of protein from the optical density. The molecular extinction coefficients of the original pure dyes differ widely (Table I ; see also Miller, Sapp and Miller, 1948 ; and Cilento, Miller and Miller, 1956) owing largely to differences in the ratio of azonium (—N$^+$H=N—) to ammonium (—N$^+$HMe$_2$) tantomers, of which the ratio $E_{520}/E_{325}$ gives a rough measure (Sawicki, 1957). For example, under the conditions of measurement used in the present paper, 3'-MeDAB and 2'-MeDAB had molecular extinction coefficients at 520 m$\mu$ of 44,400 and 10,400, and $E_{520}/E_{325}$ ratios of 5 and 0·6 respectively. However, the $E_{520}/E_{325}$ ratios of their derivatives bound to liver proteins were found to be 1·2 and 0·8. This observation that the difference between these values is far less than in the case of the original unbound dyes suggests that there may also be less difference between the molecular extinction coefficients at 520 m$\mu$ of the bound dyes, so that the $E_{520}$ values may give an approximate measure of the extent of binding of the various dyes.

TABLE I.—*Molecular Extinction Coefficients at* 520 *m$\mu$ of some Aminoazo Dyes in* 7N *HCl-ethyl Alcohol* (5 : 4)

| | |
|---|---|
| 3'-MeDAB | 44,400 |
| 2-MeDAB | 49,600 |
| DAB | 42,800 |
| 2'-MeDAB | 10,400 |
| DEAB | 15,700 |
| AB | 26,600 |

The molecular extinction coefficients of aminoazo dyes are also influenced by demethylation of the —NMe$_2$ group. Sawicki (1957) found that the molecular extinction coefficients at 520 m$\mu$ of some dyes with an unsubstituted amino group were several times smaller than the molecular extinction coefficients of their monomethylamino and their dimethylamino derivatives. Since N-demethylation is one of the major metabolic reactions of aminoazo dyes in the body (Miller and Miller, 1953 ; Conney *et al.*, 1957 ; Matsumoto and Terayama, 1961), this is another factor which may influence the extinction coefficients of the bound dyes. As pointed out by Dijkstra and Joubert (1961), the difference in the wavelengths of maximum absorption of the dye bound to liver and serum proteins of the same rat may be a reflection of a difference in the extent of N-demethylation in these two cases.

Because of these factors, care must be taken when interpreting differences in the optical density of the bound dyes as differences in the number of moles of dye bound to proteins. With this reservation in mind, the results may now be considered.

The results of the bound dye estimations are given in Table II. The maximum levels in both the liver and the serum were found after about 38 hours, except in the case of 2-MeDAB where it appeared at about 50 hours. The levels of bound dye in the liver after the present intragastric administration of 3′-MeDAB were at all times comparable to those found by Gelboin, Miller and Miller (1958) after a single intraperitoneal injection.

The maximum density (E) of bound dye in the liver varied with the different compounds, being highest (about 0·2) in the case of the most carcinogenic, 3′-MeDAB, less for the moderately carcinogenic DAB (0·07) and lowest in the case of the weakly carcinogenic 2′-MeDAB (0·05) and the non-carcinogens DEAB (0·05) and AB (0·05). In the case of the very weak carcinogen, 2-MeDAB, appreciable binding was observed in the liver, with an optical density at the maximum of about 0·13.

The binding of dye to the serum proteins followed the same time course as that of the binding of dye to the liver proteins, but the amounts of dye bound to the former were always lower than in the case of the latter. With 3′-MeDAB, DAB, 2′-MeDAB, DEAB, and AB, the averages of the ratios of bound dye absorbance per unit weight of protein in the serum to that in the liver were 0·47, 0·50, 0·49, 0·53 and 0·51 respectively. With 2-MeDAB, however, the ratio was considerably lower, namely 0·19 so that the low level of binding to serum proteins was more in line with its low carcinogenicity than its high binding to liver proteins.

The result of some determinations of free (alcohol-extractable) dye in the liver and serum of rats dosed with 3′-MeDAB and 2-MeDAB are presented in Table III. These show that the levels of free dye in the serum and the liver ran parallel. In both cases little free dye could be detected during the first 10 hours after administration ; dye levels reached a maximum after 20 to 40 hours, and declined to very low levels again after 88 hours. Comparison of these results with those in Table II shows that the time interval between the appearances of free and bound dye in the liver was very small and that the bound dye maximum was found when the free dye level decreased. This suggests that the binding process was relatively rapid.

Table III also shows that the ratio of optical density of free dye in the serum to that in the liver was greater than unity in the case of 3′-MeDAB, whereas it tended to be less than unity for 2-MeDAB. Thus in the case of both free and bound dyes 3′-MeDAB gave a higher ratio of optical density in the liver to that in the serum than 2-MeDAB.

Experiments in which 0·6 mg. of 3′-MeDAB or 2-MeDAB was added to 4 ml. of blood containing 0·2 per cent potassium oxalate, and allowed to stand for 16 hours at 37°C., showed that no *in vitro* formation of bound dye occurred in the blood.

### DISCUSSION

The results obtained in the present study with a single large dose of dye are similar to those of Miller *et al.* (1949) who fed a low level of dye continuously in that, assuming that the molecular extinction coefficients of the different bound

TABLE II.—*Protein-bound Azo Dye in the Serum and the Liver of Rats After Intragastric Administration of Various Aminoazo Dyes*

$\frac{\text{Dye in serum}}{\text{Dye in liver}}$ (optical density, E, per 50 mg. protein)

| Time after dosing (hours) | 3′-MeDAB | 2-MeDAB | DAB | 2′-MeDAB | DEAB | AB |
|---|---|---|---|---|---|---|
| 1 | 0·010/0·017 = 0·59 | | | | | |
| 2 | 0·009/0·014 = 0·64 | | | | | |
| 3 | 0·010/0·028 = 0·36 | | | | | |
| 4 | 0·018/0·016 = 1·13 | | | | | |
| 6 | 0·012/0·024 = 0·50 | | | | | |
| 10 | 0·016/0·032 = 0·50 | | | | | |
| 14 | 0·049/0·102 = 0·48 | 0·001/0·013 = 0·08 | 0·012/0·025 = 0·48 | 0·016/0·029 = 0·55 | 0·010/0·018 = 0·56 | |
| 19 | 0·054/0·121 = 0·45 | 0·010/0·042 = 0·24 | | | | |
| 20 | 0·073/0·216 = 0·34 | 0·007/0·022 = 0·32 | 0·025/0·036 = 0·69 | 0·014/0·027 = 0·52 | 0·026/0·040 = 0·65 | |
| 26 | 0·053/0·125 = 0·42 | 0·010/0·074 = 0·13 | 0·025/0·066 = 0·38 | 0·020/0·035 = 0·57 | 0·024/0·050 = 0·48 | |
| 32 | 0·073/0·144 = 0·51 | 0·010/0·058 = 0·17 | 0·041/0·071 = 0·58 | 0·021/0·048 = 0·44 | 0·028/0·051 = 0·55 | |
| 38 | 0·108/0·252 = 0·43 | 0·020/0·110 = 0·18 | 0·035/0·066 = 0·53 | 0·021/0·047 = 0·45 | 0·027/0·057 = 0·47 | 0·014/0·028 = 0·50 |
| | | 0·011/0·088 = 0·13 | 0·020/0·053 = 0·38 | | | 0·021/0·043 = 0·49 |
| | | | 0·023/0·049 = 0·47 | | | 0·016/0·030 = 0·53 |
| 39 | | 0·024/0·149 = 0·14 | | | | |
| 40 | 0·073/0·241 = 0·30 | 0·016/0·096 = 0·17 | | | | |
| 42 | 0·081/0·202 = 0·40 | 0·021/0·081 = 0·26 | | | | |
| 44 | 0·057/0·179 = 0·32 | 0·017/0·108 = 0·16 | 0·032/0·068 = 0·47 | 0·014/0·035 = 0·40 | 0·019/0·042 = 0·45 | |
| | 0·036/0·131 = 0·27 | 0·019/0·138 = 0·14 | | | | |
| 50 | | 0·028/0·117 = 0·24 | | | | |
| 51 | | 0·032/0·121 = 0·26 | | | | |
| 64 | 0·044/0·136 = 0·32 | | | | | |
| 65 | | 0·024/0·127 = 0·19 | | | | |
| 88 | 0·033/0·108 = 0·31 | 0·010/0·055 = 0·18 | | | | |
| | 0·030/0·112 = 0·27 | | | | | |
| 89 | | 0·029/0·117 = 0·25 | | | | |
| Average ratio | 0·47 | 0·19 | 0·50 | 0·49 | 0·53 | 0·51 |

TABLE III.—*Free Azo Dye in the Serum and the Liver of Rats After Intragastric Administration of 3′-MeDAB and 2-MeDAB (optical density, E, per 50 mg. protein)*

| Time after dosing (hours). | 3′-MeDAB | | 2-MeDAB | |
|---|---|---|---|---|
| | Serum. | Liver. | Serum. | Liver. |
| 1 | 0·07 | 0·06 | — | — |
| 1 | 0·14 | 0·09 | — | — |
| 2 | 0·05 | 0·08 | — | — |
| 3 | 0·05 | 0·27 | — | — |
| 4 | 0·20 | 0·09 | — | — |
| 6 | 0·05 | 0·04 | — | — |
| 10 | 0·05 | 0·01 | — | — |
| 19 | 1·52 | 1·33 | 0·14 | 0·25 |
| 38 | — | — | 0·75 | 0·97 |
| 40 | 0·85 | 0·36 | 0·23 | 0·44 |
| 42 | 2·05 | 0·75 | 0·16 | 0·28 |
| 44 | 0·16 | 0·03 | 0·31 | 0·30 |
| 50 | — | — | 0·29 | 0·09 |
| 51 | — | — | 0·42 | 0·36 |
| 88 | 0 | 0·14 | 0 | 0·08 |

dyes are approximately the same, the extent of binding of a dye to the proteins of the liver parallels its carcinogenicity. In both Miller's and the present work, the weak carcinogen, 2-MeDAB, was an exception to this rule.

After continuous feeding it was found that although the amount of 2-MeDAB bound in the liver eventually exceeds those formed in rats fed the most actively carcinogenic members of the series, the time required for the dye concentration to reach a maximum was considerably longer for 2-MeDAB than for the more potent carcinogens. The present work suggests that with a single dose also a high concentration of bound dye in the liver persists longer in the case of 2-MeDAB than in that of 3′-MeDAB. Further work, however, would be necessary to confirm this.

Miller *et al.* (1949) consider that the time interval after which maximum binding of a dye occurs in the liver following continuous feeding is more closely related to its carcinogenicity than the actual level of dye attained, since the fall in dye concentration on prolonged feeding is a sign of the deletion of the dye-binding proteins. This hypothesis does not, however, explain why binding of active carcinogens should result in deletion of the proteins more readily than binding of apparently similar amounts of weak carcinogens, such as 2-MeDAB.

From this point of view, the finding that the binding of 2-MeDAB to serum proteins is more in line with its low carcinogenicity is of considerable interest. Since no *in vitro* formation of bound dye occurred in the blood, the binding of dye to serum proteins presumably occurs in the liver, possibly at the time of their synthesis. It thus appears that the liver differentiates between serum and tissue proteins in respect of the extent or mode of dye binding.

It would be interesting to see whether the relative extent of binding of 3-MeDAB to liver and serum proteins is similar to that of 2-MeDAB which it resembles in giving a higher level of bound dye in the liver on continuous feeding than is expected from its low carcinogenicity (Miller *et al.*, 1949). If this is the case, it might provide a clue to the critical locus of action of the carcinogenic azo dyes on proteins in the liver.

SUMMARY

The extent of binding of dye to proteins was determined in the liver and serum of rats after administration by stomach tube of a single dose of 50 mg. of various aminoazo dyes. Free (i.e., alcohol-extractable) dye was also determined in the case of 3′-MeDAB and 2-MeDAB. The free dye levels in both serum and liver were low during the first 10 hours, rose to a maximum after 20 to 40 hours, and then declined. The appearance of the bound dye in the liver and in the serum followed parallel courses. In both, the maximum dye level was reached after about 40 hours, when the level of free dye decreased. The extent of binding of the various dyes to proteins in the liver paralleled their carcinogenicity, except in the case of 2-MeDAB, which is a weak carcinogen, but which was strongly bound. In the case of the other dyes, namely 3′-MeDAB, DAB, 2′-MeDAB, DEAB, and AB, the ratio of bound dye (expressed in optical density units) per unit weight of protein in the serum to that in the liver averaged 0·5, whereas with 2-MeDAB it was only 0·19. The binding of 2-MeDAB to serum proteins is therefore more in line with its low carcinogenicity than its binding to liver proteins.

The authors wish to thank Dr. H. M. Schwartz for valuable criticism, and Mr. J. J. Dreyer and his staff of the Physiology Section of the National Nutrition Research Institute for their kind co-operation in providing and housing all the animals.

This project was sponsored in part by the National Cancer Association of South Africa.

### REFERENCES

CILENTO, G., MILLER, E. C. AND MILLER, J. A.—(1956) *J. Amer. chem. Soc.*, **78**, 1718.

CONNEY, A. H., BROWN, R. R., MILLER, J. A. AND MILLER, E. C.—(1957) *Cancer Res.*, **17**, 628.

DIJKSTRA, J. AND JOUBERT, F. J.—(1961) *Brit. J. Cancer*, **15**, 168.

GELBOIN, H. V., MILLER, J. A. AND MILLER, E. C.—(1958) *Cancer Res.*, **18**, 608.

MATSUMOTO, M. AND TERAYAMA, H.—(1961) *Gann*, **52**, 239.

MILLER, E. C. AND MILLER, J. A.—(1947) *Cancer Res.*, **7**, 468.

*Iidem*, SAPP, R. W. AND WEBER, G. M.—(1949) *Ibid.*, **9**, 336.

MILLER, J. A. AND MILLER, E. C.—(1948) *J. exp. Med.*, **87**, 139.—(1953) *Advanc. Cancer Res.*, **1**, 339.

*Idem*, SAPP, R. W. AND MILLER, E. C.—(1948) *J. Amer. chem. Soc.*, **70**, 3458.

SAWICKI, E.—(1957) *J. org. Chem.*, **22**, 621.